# Sunshine

**Published:** 1880-07

**Authors:** 


### SUNSHINE.

This journal has very frequently advocated the power of moral medicines as being more efficient in many cases than any physic of the apothecary; so in this article we will let material, out-door sunshine alone, and say something of the sunshine of the heart and hearth; of its power to insure a new life and activity when physical toil has used up the vital energies, and when insidious disease has sapped the powers of life, and left the body a mere wreck of what it was.

But a year or two, or a month or two, sometimes even a few weeks of no sunshine in the heart, have been all sufficient to lay the body in the grave to be at rest at last. There are some men, spoonies, who look as if they had never smiled, there is a pitiful sadness, with an unmistakable expression of feature, a kind of hopelessness, as if they were kept under all the time at home. They don't exactly die; it's a great pity they didn't; they seem to have got used to it, and settled down in a state of sorrowful submission; they hadn't sense enough to maintain their liberties, nor energy enough to run away when every thing was lost.

There are other men, brave, indomitable; who live above the present; who having found themselves "in a fix," by having made a grand mistake in marriage, have made a virtue of necessity, and have proudly determined to "endure," to the end of the chapter. At the same time, there is a settled sadness on the features when at rest, showing plainly that there is no "sunshine" at home.

But the sight that pains us most, is that in Broadway, of young women and those of maturer years, in whose faces it is plainly seen there is no sunshine at home; but the skeleton of a step-mother; of a trifling husband; or of one who has no sympathies; nothing in common with the woman of his choice: she, refined, educated, with cultivated tastes, of sensitive instincts; he, ignorant, debased, brutal.
He was rich; she poor; hence the tale of sadness; a home without any sunshine.

There is a young girl, not very well dressed; she would be handsome if she were; she walks as if it were done mechanically; as if there was no object ahead; as if she were going toward home, but did not care whether she ever got there or not; there is no spring, no elasticity in her step, but as if an iron weight were attached to each heel. There is certainly no sunshine under the roof which shelters her. Perhaps she has a drunken father; a brother who is a disgrace to the family; or her mother may be her skeleton, by having no feelings in common with her; thwarts her in all her undertakings, in all her plans; always disparaging, always finding fault, always giving directions; never satisfied with the manner in which any thing is done, and whose whole life is a dirge. Poor girl! a little sunshine at home, how it would lighten up her countenance, brighten her face, and make a greater change in her whole moral character, than any "sunshine" which Mrs. Dall describes, could make on the physical nature.

But what a light and life and genial warmth must be in the home of that woman who writes so well of the out-door sunshine; there must be an atmosphere of moral loveliness there, the mere thought of which actually "makes our mouth water," and gives us an earnest, an inappeasable longing to peep in upon them, just a moment of any hour of any day or evening; the daughters—how lady-like, how affectionate; the sons—how joyous and how manly; and the husband, happy dog, looking on with quiet satisfaction; first upon the girls, then on the boys, and anon instinctively resting his eyes with fond satisfaction on the composed and heaven-like features of the fond wife of his bosom, as the "author and giver of them all," next to Him who rules above. Let every thing that has [illegible] most distant resemblance to a contemptible whine, to a devilish fault-finding, to a brutal boorishness and to a narrow minded and degrading selfishness, be considered as emanations from that pit of darkness, where fiends and furies dwell; then shall light be in every family dwelling; cheerfulness in every face

There are men everywhere who are daily crushing out the hearts of the women who left happy homes to nestle confidingly in their bosoms; not always with deliberate design, but by a thoughtless inattention in the exhibition of those sympathies on which most women feed as flowers do on water. There are women, too, who have so much of wormwood and of gall in their composition that their first morning's utterance is a whine or a growl; who never, by any chance, sit down to the family table without a complaint; "pecking" first at one child, then at another; or servant or neighbor, or acquaintance or friend; who never enter a room without some exhibition of querulousness or dissatisfaction. We were witness of an extraordinary exemplification of this hateful feature in the character of a mother, about two years ago, in a family who had not been a great while in New-York. Some four or five, may be more children were in the parlor; some playing, some reading, some building mimic houses on the floor; we were contemplating the scene at the time, as one fit for a painter to transfer to canvas; there was a deep satisfaction, or a more uproarious gladness in every countenance, when the door opened and the mother entered; a thin, bilious, scraggy, hatched-faced woman, with an apparent spinal deformity, which almost doubled her up, as her head was not much higher than the door-lock. As instantaneous as a flash of lightning, every voice was hushed; all play was suspended, and there was the silence of the grave; the woman looked around the room, said not a word, and withdrew. As soon as the door closed, a little boy of five, straightened himself up as he sat on the floor, drew a long breath and exclaimed in a tone of surprise: "I thought mamma had come to kick up a fuss." As if it was one of the strangest of occurrences that she should come into a room where her children were, without saying or doing something calculated to bring a cloud over the household. It is related of a merchant's widow, somewhere in Brooklyn, that she was of such a fault-finding and querulous nature, that her only son had not slept in the house for two years! The two daughters, who had reached womanhood, exclaimed one day to a lady friend of ours whose face was always a sun: "O Mrs. P.! if mother was only like you, how happy we and brother would be." But the father, where was he—in his grave! the acknowledged victim of his wife's habitual ill-nature and fretfulness.

And what shall we say of the husband and father, who comes home with a scowl; who brings a cloud with him which darkens the whole household the moment he enters the door, who frets and complains at every thing; whom nothing can please, whom nobody can satisfy? There are such men, and even worse. The cases above are rare; one, it may be in a decade or in a hundred thousand; but perhaps almost all have the germs of these undesirable traits, which ought to be watched against every hour of our existence, lest they might grow before we are aware of it, to unmanageable proportions; but this is only half our duty; we should sedulously cultivate all opposite qualities, that a pure and a true affection and a loveliness of disposition and temperament should so impregnate the whole character, that the household should be only the ante-chamber of heaven!

